# Governing Integrated Health and Social Care: An Analysis of Experiences in Three European Countries

**DOI:** 10.5334/ijic.7610

**Published:** 2024-02-07

**Authors:** Josephine Exley, Rebecca Glover, Martha Mccarey, Sarah Reed, Anam Ahmed, Hubertus Vrijhoef, Tommaso Manacorda, Concetta Vaccaro, Francesco Longo, Ellen Stewart, Nicholas Mays, Ellen Nolte

**Affiliations:** 1 Policy Innovation and Evaluation Research Unit, Department of Health Services Research and Policy, London School of Hygiene and Tropical Medicine, 15–17 Tavistock Place, London WC1H 9SH, UK; 2 The Nuffield Trust, London, UK; 3 Panaxea, Den Bosch, The Netherlands; 4 Fondazione CENSIS, Rome, Italy; 5 Department of Social and Political Sciences at UniversitàBocconi, Milan, Italy; 6 School of Social Work & Social Policy, University of Strathclyde, Glasgow, UK; 7 School of Social and Political Science, University of Glasgow, Glasgow, UK

**Keywords:** integrated health and social care, governance, health system and services research, health financing, comparative policy analysis

## Abstract

**Purpose:**

Achieving greater health and social care integration is a policy priority in many countries, but challenges remain. We focused on governance and accountability for integrated care and explored arrangements that shape more integrated delivery models or systems in Italy, the Netherlands and Scotland. We also examined how the COVID-19 pandemic affected existing governance arrangements.

**Design/methodology/approach:**

A case study approach involving document review and semi-structured interviews with 35 stakeholders in 10 study sites between February 2021 and April 2022. We used the Transparency, Accountability, Participation, Integrity and Capability (TAPIC) framework to guide our analytical enquiry.

**Findings:**

Study sites ranged from bottom-up voluntary agreements in the Netherlands to top-down mandated integration in Scotland. Interviews identified seven themes that were seen to have helped or hindered integration efforts locally. Participants described a disconnect between what national or regional governments aspire to achieve and their own efforts to implement this vision. This resulted in blurred, and sometimes contradictory, lines of accountability between the centre and local sites. Flexibility and time to allow for national policies to be adapted to local contexts, and engaged local leaders, were seen to be key to delivering the integration agenda. Health care, and in particular acute hospital care, was reported to dominate social care in terms of policies, resource allocation and national monitoring systems, thereby undermining better collaboration locally. The pandemic highlighted and exacerbated existing strengths and weaknesses but was not seen as a major disruptor to the overall vision for the health and social care system.

**Research limitations:**

We included a relatively small number of interviews per study site, limiting our ability to explore complexities within sites.

**Originality:**

This study highlights that governance is relatively neglected as a focus of attention in this context but addressing governance challenges is key for successful collaboration.

## Introduction

Many countries are seeking to better integrate care at the community, primary and secondary care interfaces, and between health and social care (referred to as long-term care in some contexts) to meet increasingly diverse health and care needs [1]. The need for more effective service integration has become ever more important in the context of the COVID-19 pandemic [2]. While unleashing rapid service transformation in some areas such as acute care, the pandemic highlighted, and exacerbated, a lack of coordination between the health and social care sectors, and between primary and specialist care, in many countries, which made an effective response more problematic [3].

A key challenge to achieving better integration is the complexity involved in any strategy that seeks to bring together different service sectors within health care and between health and social care, and address the varied set of interests and priorities of those involved in the delivery and financing of health and social care services [4]. Existing work has highlighted the problems arising from disjointed planning, lack of oversight and unclear lines of accountability, leading to service duplication and gaps, which can undermine the effectiveness and efficiency of service delivery locally [5]. Other evidence has highlighted the challenges of establishing novel governance arrangements at local or regional levels, which may be at odds with the national policy and regulatory frameworks [6]. Understanding and addressing these challenges will be key for successful collaboration between the various actors involved.

This study focused on governance and accountability for integrated health and social care and explored arrangements in Italy, the Netherlands and Scotland at regional and national levels that influence, directly or indirectly, more integrated delivery models or systems. Conducted during the COVID-19 pandemic, it further sought to capture how the pandemic affected existing governance arrangements and shaped health and care service delivery in response.

## Methods

We undertook a multiple-country analysis using a case study approach involving document review and interviews [7]. We considered multiple countries and multiple cases (‘study sites’) within each country to generate a broad understanding of factors that influence governance and accountability arrangements of integrated service delivery systems within each country.

### Conceptual framework

We drew on the transparency, accountability, participation, integrity and capacity (TAPIC) framework developed by Greer et al., Table 1 [8]. The framework identifies and defines five mutually exclusive attributes of governance that influence “the kind and consequences of decisions a system makes” (p. 16). In line with Greer et al., we defined governance as the “process and institutions through which decisions are made and authority in a [given setting] is exercised” (p. 28).

**Table 1:** TAPIC framework for analysing ‘good’ governance.


ATTRIBUTE	DEFINITION

Transparency	Institutions make decisions and their grounds clear

Accountability	Mechanisms for giving an account of one’s actions and being held to account for those actions/explanations are clear

Participation	Affected parties have access to decision-making power

Integrity	Processes of representation, decision-making and enforcement are clearly specified

Capacity	Necessary expertise and resources to develop policy are available


*Note*: Adapted from Greer et al. [8].

### Country and study site selection

Country selection was guided by: (i) experience of innovative approaches to service delivery that seek to bring together different sectors, in particular, health and social care; (ii) similarities in health system functioning such as operating a primary care gatekeeping system, offering most health services free at the point of use, or having municipalities responsible for overseeing (parts of) social care; and (iii) authors’ knowledge from prior work in the field of service coordination and integration and through involvement in international advisory groups and networks [1,5,9,10]. Table A-1 in the appendix provides an overview of the principles of the health and social care systems in each country.

We selected ten study sites across the three countries (3 in Italy, 4 in the Netherlands and 3 in Scotland), Table 2. An inherent challenge in any (international) study of integrated care is the many different ways in which integrated care has been, and is being, conceptualised and interpreted [11]. Therefore, study site selection was informed, mainly, by our co-authors with deep knowledge of individual country contexts and what is considered ‘integrated care’ in those contexts (ES, HV, FL), as well as document review [7]. To maximise variation, we selected sites which present different approaches to integration and at different scales.

**Table 2:** Overview of study sites.


STUDY SITE	DESCRIPTION

Italy	

Azienda Zero (AZ), Veneto	AZ is a regional governance body, which centralises key functions previously carried out by local health authorities, including procurement, human resources, legal services and cash-flow management, and quality of health and social care in Veneto region. The nature and scope of its functions are set out in regional legislation.

Chronic care (Presa in carico del paziente cronico, PiC), Lombardy	PiC is a new approach to integrated management of chronic care, in which a care manager (GP or paediatrician) develops individual care plans (*piano di assistenza individuale*, PAI) and takes responsibility for coordinating care. Managing bodies act as the primary provider of services, either directly or through contracted partners. The PiC incentivised the creation of GP cooperatives as managing bodies.

Houses of health (*Casa della salute*, CdS), Emilia-Romagna	CdS (also referred to as ‘community health centre’ or ‘medical home’) oversee access to and the provision of health and social care services locally. They coordinate primary care with other service providers, including specialist and inpatient care, develop diagnostic and integrated care pathways along with prevention programmes, and manage chronic conditions through primary and specialist care integration. The region sets out the general framework and goals for the model, which the local health authorities adapt to their specific local context.

Netherlands	

Buurtzorg	Buurtzorg is a not-for-profit social enterprise home care company in which self-governing teams of nurses provide all aspects of home care, from personal care to more complex clinical care, in neighbourhoods across the Netherlands to provide more coordinated care.

Rotterdam Stroke Service (RSS)	RSS is the largest integrated stroke care network (‘stroke care chain’) in the Netherlands, formed by 17 health care organisations that work together to organise care for stroke patients during the acute, rehabilitation and chronic phase of patient care. The RSS is divided into seven sub-chains around each participating hospitals.

Sustainable Coalitions (*Duurzame coalitie*)	Sustainable Coalitions are long-term collaborations between the health insurer CZ and service provider organisations seeking to develop and implement innovative care approaches for defined populations. The aim is to shift from negotiating yearly contracts on price and volume to longer-term contracts of up to ten years based on a contract price. The Sustainable Coalitions are based on the principle of ‘co-creation’, aiming to develop a shared vision between the insurer and provider organisation(s).

ZIO (Zorg in Ontwikkeling)	ZIO is a primary care organisation (‘care group’) supporting general practitioners, physiotherapists and dieticians in the Maastricht-Heuvelland region to provide person centered-care for various target populations, including those with chronic conditions, older people, and people with mental health problems. ZIO is the main primary care contractor in the region and responsible for negotiating contracts with the health insurer on behalf of the care group as well as sub-contracting individual providers or provider organisations within the care group.

Scotland	

Dumfries and Galloway Integration Joint Board (IJB)	Dumfries and Galloway Integration Joint Board brings together NHS Dumfries and Galloway (regional health board) and the Dumfries and Galloway Council (local authority). The services delegated to the IJB include all adult health and social care for the region. Dumfries and Galloway IJB is the only IJB in Scotland with delegated authority for acute hospitals.

East Ayrshire Integration Joint Board (IJB)	East Ayrshire Integration Joint Board brings together NHS Ayrshire and Arran (regional health board) and East Ayrshire Council (local authority). The services delegated to the IJB include public health, nursing, children, young people and criminal justice services, social work for adults, mental health services, care home services, community care assessment teams and occupational therapy among others.

Highland Integration Partnership	Highland Integration Partnership brings together NHS Highland (regional health board) and Highland Council (local authority). Highland is the only area that adopted the Lead Agency model whereby NHS Highland has responsibility for adult health and social care services and Highland Council has responsibility for children’s health and social care services.


*Note*: Authors’ compilation based on country reports (available upon request).

### Data collection

#### Document review

We used a generic data collection template to capture information on the governance and accountability arrangements within study sites. We reviewed the published and grey literature through an iterative search of PubMed and Google Scholar, and governmental and non-governmental agencies and organisations with a remit in health service and system organisation, financing, and governance in the countries under review. We included documents in English, Dutch, and Italian. The material formed the basis of detailed country reports (available from the authors upon request) and we extracted key characteristics of study sites to provide context for the key informant interview analysis presented in this paper.

#### Key informant interviews

We conducted interviews with key informants involved in the organisation, governance or delivery of local health and social care in study sites and at the national level using a combination of purposive and ‘snowball’ sampling. Participants were identified from organisations’ websites, the authors’ professional networks and recommendations from other study participants.

Potential participants were invited by e-mail and provided with background information. Interviews used a topic guide informed by the TAPIC framework [8] and tailored to participant role, exploring decision-making processes and communication, lines of accountability, role definition and responsibilities, public participation, and capacity to implement change, along with impacts of the COVID-19 pandemic on decision-making processes within local systems. Topic guides are available upon request.

All interviews were carried out using the video platforms Zoom or Microsoft Teams between February 2021 and April 2022, in English, Dutch or Italian. They lasted 30–60 minutes, were audio-recorded upon consent, translated to English (Dutch and Italian interviews) and transcribed verbatim.

We conducted 35 interviews, Table 3. To maintain confidentiality, we report participants’ country (IT = Italy, NL = Netherlands, SCT = Scotland) and their level in the system (N = national or C = study site).

**Table 3:** Overview of interviews by country.


COUNTRY	ITALY	THE NETHERLANDS	SCOTLAND

National level (N)	2	2	4

Study site 1 (C1)	4	3	2

Study site 2 (C2)	3	2	2

Study site 3 (C3)	3	3	1

Study site 4 (C4)	n/a	4	n/a

Total	12	14	9


### Analysis of key informant interviews

Interviews were analysed thematically using deductive and inductive coding. The initial coding frame was developed by JE and RG using the five elements of the TAPIC framework as a pre-specified thematic framework. However, during the analytical process we found considerable overlap between attributes of TAPIC, and we therefore revised the coding frame based on themes identified inductively from interviews. The coding frame was developed further and finalised by involving all researchers who had conducted interviews (JE, RG, MM, SR, AA, HV, TM) and who read a sub-set of interviews from countries other than those they had conducted interviews in. All interviews were coded against the agreed framework in Excel by members of the research team. The key themes were finalised by grouping and re-grouping the codes and informed by discussion between researchers involved in interviews and the wider author team.

### Ethical approval

The study received ethical approval from the Observational/Interventions Research Ethics Committee at the London School of Hygiene & Tropical Medicine (Ref. 17988).

## Findings

Table A-2 summarises key characteristics of the ten study sites based on our document review. Study sites represent a spectrum of innovative integrated care approaches that reflect the general regulatory and policy framework within which those involved operate, illustrating a combination of ‘bottom-up’ and ‘top-down’ processes to driving integration. In the Netherlands, bottom-up voluntary arrangements typically involve different types of providers, and health insurers are playing an increasingly central role in facilitating new ways of working using innovative forms of contracting [12]. For example, the ‘Sustainable Coalitions’ include small scale, albeit rapidly evolving, long-term agreements between payers (health insurer) and providers (primary, secondary and mental health care) to improve the quality and accessibility of services for defined populations.

At the other end of the spectrum sits Scotland, where health and social care integration has been mandated through the 2014 Public Bodies (Joint Working) (Scotland) Act. It placed a legal duty on NHS Boards and Local Authorities to pool budgets and jointly plan and commission certain health and social care services [13]. It led to the formation of 31 Integration Authorities, which delegate responsibility for health and social care to new bodies, the Integrated Joint Boards (IJBs) (with one exception, where responsibility is delegated to a ‘Lead Agency’, see Table A-2). Italy sits somewhere in-between the Netherlands and Scotland, with regional governments leading efforts to improve integration through regional reform within a national legislative framework (which is often informed by regional reform efforts) [14].

We identified seven themes reported to have helped or hindered integration efforts locally: integration of policies and institutional arrangements at national level; clarity of lines of accountability; national government understanding of local context; engaged local leadership; knowing whether integration makes a difference; integrated financing; and the impact of COVID-19.

### Integration of policies and institutional arrangements at national level

There was agreement among participants from all countries that there was a disconnect between what national governments aspire to achieve, in terms of a wider conceptualisation of health and care integration, and national governments’ own efforts to implement the vision. A siloed approach in national policies was reported to be commonplace and seen to undermine integration efforts locally. In practice, this may manifest in various ways, such as organisations, programmes, and health services that vary in their mandate at local level.

“We are actually asking the field to collaborate across domains, while we as a government actually do that too little … we are still very [sector]-oriented. So, you see that all those directorates […] and all those implementing organisations thus set up their own programmes, which may be based on the same philosophy but still are slightly different, or even sometimes contradict each other.” (NL_N_1)

In Scotland, while the 2014 Act provides a national vision, the existence of several directorates at national level covering health, local government and integration, meant that Integration Authorities could receive different directions from each directorate. This lack of strategic integration was perceived by some to weaken collective integration efforts.

In Italy, similar issues were reported to play out at the regional level, with responsibility for different aspects of care assigned to different directors, leading to fragmented governance.

“The Milan ATS [one of the 8 planning and commissioning health agencies in Lombardy] has a managing director, a health care director, a social care director, and a primary care department director for each ATS. Now […] they’re talking about appointing district directors who are supposed to coordinate the activities of the community care facilities [responsible for integrating health and social care] … In my opinion here there is really a problem of [lack of] governance. If you look at the new Lombardy regional reform, you see that governance is fragmented in such a way that in the end no one is accountable for anything.” (IT_C1_2)

Study participants acknowledged that developing new ways of working takes time; integration therefore needed to be viewed as a long-term policy goal. Respondents in Italy and the Netherlands specifically commented that the role of national government should be to create and sustain a long-term vision to provide a coherent and consistent policy framework that transcends political lines and electoral cycles.

### Clarity of lines of accountability

Independent of the nature of governance arrangements in place, the perceived lack of alignment at national level blurred the lines of accountability, which, in turn was seen as a risk to integration locally.

For example, in Scotland, the 2014 Act mandating the formation of Integrated Authorities was perceived to be *“some of the most progressive social care legislation around”* (SCT_N_3), providing a unified national vision. Yet, study participants suggested that this had not translated into effective local collaboration, noting that the reforms had been overly bureaucratic, resulting in a complex governance landscape and a lack of clarity in accountability arrangements and decision-making processes. Participants reported instances of circularity between the different local actors, whereby Integrated Joint Boards are accountable to both NHS Boards and Local Authorities, and vice versa. This created “*an odd situation where on one hand you tell me what to do and then on the other wow, you’re doing what I tell you to do”* (SCT_C2_1).

A degree of circularity of roles and responsibilities was also observed in the Netherlands, with a participant from one of the health insurers highlighting their role in engaging in collaboration with providers to develop new ways of working, taking the role of an ‘integrator’, while at the same time being responsible for holding providers to account. A particular issue arising in the Netherlands was the lack of clarity around responsibilities for payment of services, especially at the boundary between health and care services.

The involvement of local government was seen by some participants in Italy and Scotland to create challenges where elected officials’ priorities do not match those of the health and social care system.

“[I]t is evident that there is an information asymmetry between us – managing directors and regional health department – and the mayors. This is a problem, because then the mayor responds to citizens’ associations, to his constituents [gives example of a specific election promise]. The mayor does not listen [to managing directors], politics does not listen, politics is convinced to be right because it responds to associations, citizens and therefore… So, this is a big problem.” (IT_C2_1)

Study participants from Italy and the Netherlands highlighted the potential value of ‘neutral’ or independent technical bodies to take on management or oversight roles for (e.g., Azienda Zero in Veneto) or on behalf of (e.g., ZIO care group in the Netherlands) providers, acting as liaison with the wider system. The neutral position was seen to allow organisations to transcend the day-to-day challenges of service commissioning and/or delivery and provide strategic guidance and leadership. Moreover, one participant explained that this perception of impartiality can foster ‘pleasant’ and ‘comfortable’ relationships among technocrats, suggesting that trustworthiness can more readily emerge when organisations are delinked from political agendas.

“[T]he most interesting aspect of the role of Azienda Zero, which is not to do activities on behalf of ASLs [health authorities], or in part on behalf of ASLs, but above all to make knowledge available to all ASLs in a sort of virtual platform, a moment of information sharing, also aligning behaviours”. (IT_C3_2)

### National government understanding of local context

There was widespread agreement across participants from all countries that irrespective of whether service integration efforts were driven locally or nationally, support from national government requires a good understanding of the local context to be effective. Where this was lacking, there was a reported disconnect between national policy ambitions and the realities on the ground, which may lead to *“a big gap, I think, between the daily practice and the policy-makers, from governmental perspective”* (NL_C3_1), which can result in frustration among both local and national actors.

In Scotland, the 2014 Act was not considered sufficient to overcome competing incentives between actors locally. In Italy, the regional approach to health reform was seen by some participants as reinforcing, rather than countering, inequalities across the country. There appeared to be a strong desire among Italian study participants for a more strategic framework at national level to guide regional integration efforts. There was some expectation that new national standards laid out in the Italian national COVID-19 recovery plan will provide greater unity and lead to actions to reduce inequalities between regions [15].

Like Italy, the role of the Dutch national government was reported as providing oversight and to develop the wider regulatory and policy framework for the health and social care system. In contrast to Italy and Scotland, the Dutch government was seen to have so far avoided direct legal measures to enforce integration, instead favouring incremental change to the existing system.

“You want to connect with the existing structures, you do not want to place new responsibilities […] that goes against the existing frameworks […], but you must indeed ensure that parties cannot run away from their responsibility because you also build in some kind of legitimacy.” (NL_N_1)

Some Dutch interview participants were resistant to the idea of further national government involvement, favouring a more bottom-up approach. The freedom and flexibility to adapt to local contexts was seen as key to the success of integration efforts.

### Engaged local leadership

Within study sites, local leadership was identified as critical to building a common vision across participating organisations, driving engagement and advancing the integration agenda: “*you need someone who’s in charge and you need someone who’s keeping this vision alive”* (NL_C3_1). Absent or changing leadership was seen to negatively affect morale, perceptions of success, and, possibly, care provision itself.

Stability in leadership appeared to be a particular challenge in Scotland, with study participants describing high levels of turnover among senior leaders. Leadership was seen to be further undermined by a perceived lack of power for chief executives to exercise their mandate, caught between the needs of NHS Boards and Local Authorities.

“For the IJB to be purely the servant of the whim of a [NHS] Board and a Local Authority, as is established in law in the Public Bodies Act… it crippled the process at birth […]. It made the Chief Officer, the meat in the sandwich of constant battles between the [NHS] Board and the Local Authority […]. There is not one Health and Social Care Partnership that has not had at least one change of Chief Officers, the majority have had two…” (SCT_N_3)

A greater clarity of authority within the Dutch study sites and the more distal relationship with national government appeared to give local leaders considerable mandate to define the strategic direction of the local system. Local leaders were seen as key players in advancing the integration agenda by, for example, pressing health insurers and national government to implement changes that enable greater integration.

### Knowing whether integration makes a difference

In all settings, national indicators to monitor integration efforts were perceived to be skewed towards acute health care, and insufficient to comprehensively capture the broader aims of integration. Scotland had introduced an integrated outcomes framework as part of the 2014 Act, yet, study participants highlighted a significant gap in social care indicators, which they attributed to the fact that while NHS Boards had a statutory requirement to report to national government, Local Authorities did not. Respondents in Italy also remarked on a political focus on acute care.

A lack of comprehensive data on social care was seen to hinder good working relationships across health and social care services and was seen to prevent more genuine collaborative decision-making. In all countries participants highlighted that there was a need to develop their own indicators to measure social care and integration components of service delivery. They discussed the need “*to be very clear about [data] application and usability”* to avoid *“data disenchantment and people are getting tired of continually providing data and not seeing any alteration to practice as a result”* (SCT_N_3). In all settings, participants considered that data should ideally be used to learn for improvement, inform decision-making, build an open culture and support the development of a common vision across organisations.

“This type of cross-domain multidisciplinary collaboration really needs to look at different monitoring mechanisms than the traditional ones we have. You don’t have to throw them overboard, but then there are simply more things to consider than in traditional monitors. And that on the one hand it seems more complicated, on the other hand, my counter-argument is always, you’ve created a kind of make-believe world if you used to only look at that one small set of that one care provider who performed one action” (NL_C1_1)

### Integrated financing

The need for a coherent and consistent vision for integration across levels was seen to be particularly pertinent for financing of services. Financing barriers were reported to be especially challenging by Dutch respondents, highlighting a complex and fragmented landscape involving multiple actors both at local (*“every organisation has its own finance system with, and the contracts, with their own health care insurers”* (NL_C2_1)) and national levels, that is, between the national insurance and the social sector. There was recognition that the successful scaling up of pilots required a coherent national financing framework: “*[T]he national government must remove the number of obstacles to enable [regions] to take that next step, but also to establish new frameworks and new instruments”* (NL_N_1). From other accounts, we also learnt that changes to the financing of services to facilitate better integration is yet to happen.

In Scotland, even though health and social care budgets are largely pooled, there was a perception that, in practice, it had not been sufficient to break the connection between the funding source (NHS or social care budget) and allocation: “*the IJB makes a decision to probably just put it back to the same place if I am absolutely honest”* (SCT_N_2). However, others argued that without legislation, efforts to share resources would have remained even more limited, since previous informal arrangements such as ‘budget alignment’ had failed to drive progress.

The failure to create truly pooled budgets in Scotland was attributed, in the main, to a lack of willingness among local key actors to ‘give up their resources’, but also the constraints imposed upon newly created Integration Authorities by the UK government’s policy of public sector financial austerity following the 2007/08 global financial crisis.

“[T]he potential for creativity was denied at birth and the constant necessity of making efficiency savings at a time of rising demand was doomed to failure.” (SCT_N_3)

The need for cost control and efficiency savings was commonly described as a main driver for and accelerator of national integrated care policies in all settings. Yet there was agreement that this had constrained what could be achieved locally and come at the expense of a focus on quality of care.

“I personally think that that [costs] is not quite the most important argument for doing things like this and it may even be the case that it only leads to higher health care costs in the long run. […] you can prevent care use, but that this will not mean that someone has lower health care costs over their lifetime, but that they live a healthier life.” (NL_N_2)

The desire for achieving cost savings and the use of targets were reported to play a central role in monitoring and evaluation. Respondents in the Netherlands highlighted challenges of estimating cost savings where impacts are intended to be realised across the health and social care systems. Instead, evaluation might more usefully focus on provider and service user assessments of whether integrated service delivery made a difference to them.

In Scotland and Italy, poor financial performance was reported to be the overriding factor for regional initiatives to be brought under national government management.

“[W]e have got an escalation framework and [NHS Boards] I suppose in the past have largely been escalated [up] for not meeting their financial targets, which is a bit one-dimensional really! […] we’ve backed off that a little bit over the course of the pandemic, and I would like us … to think … a bit more about Balance Scorecard. So money is important, but actually [so is] quality.” (SCT_N_2)

### Impact of COVID-19

In all countries, while the COVID-19 pandemic was reported to have had a major impact on service delivery, it was not perceived as a great disruptor to the overall vision for the health and social care system. If anything, the pandemic was seen to have reduced some bureaucratic processes, for example recruitment and additional funding, as reported in Scotland: *“financial assistance around COVID that has freed us up to do things”* (SCT_H_2). This was seen to have alleviated some of the challenges that Integration Authorities were facing. However, these impacts were not reported to be long lasting.

“I don’t really see [COVID-19] as a game changer. [Integration] was already something that played out in many places, there were discussions about it in many places. […] It is something that has perhaps also gained momentum since, I would say, since the financial crisis in 2008 and then in 2011. That was perhaps a more important driver than [COVID-19].” (NL_N_2)

There was a general perception that the pandemic had accelerated the implementation of policies that were underway in some form already, with respondents in Italy and Scotland highlighting a strengthened role of central government.

The pandemic was reported to have highlighted and exacerbated system fragmentation in all settings: “*all the problems and limitations all at once […] if you take away the COVID reference, these are all issues that have been discussed and needed to be addressed for many years”* (IT_N_1). There was agreement that integrated systems that build on long-standing, pre-existing relationships and operate a culture of openness based on broad participation and engagement across different stakeholders had been able to respond more rapidly and adeptly to the pandemic. In this way, COVID-19 brought existing weaknesses in health and social care systems to the fore and reinforced the value of working across traditional boundaries.

## Discussion

This study set out to explore the governance arrangements at regional and national levels that have influenced the establishment and further development of innovative, integrated health and social care delivery models or systems in Italy, the Netherlands and Scotland. All three countries have introduced policies and/or legislative frameworks over time to support integration within health care and between health and social care locally. In Italy and Scotland this has involved a more recent move to regionally or nationally prescribed or mandated instruments to integration [5], such as legislation for the formation of integrated care systems or models, while in the Netherlands, bottom-up approaches led by providers dominate, albeit within a wider enabling policy framework that allows for local experimentation of integrated care [16]. Our data highlight the complex and context-dependent nature of governance, whereby decisions made at national level impeded more successful integration locally, posing challenges that decision makers in study sites were facing.

Our findings confirm many of the difficulties of integration efforts that have previously been documented. For example, various reviews of health and social care integration in England have highlighted lack of oversight and strategic governance at the different tiers of the system along with a lack of clarity about the overarching ambition to transform health and social care to be among the key challenges and undermining more integrated service provision [17,18,19]. We found that those involved in innovative integrated care approaches in the three countries described very similar challenges.

A reported lack of consistency and a siloed approach at national and, in Italy, regional levels, resulted in blurred lines of accountability and misaligned incentives at the provider level, as well as a continued imbalance between health and social care. These issues were seen to be most acute in Scotland, where the 2014 legislation built-in competing accountabilities; increasing accountability to national government from NHS Boards while Local Authorities remained primarily accountable to their electorates, which is also a feature of the Italian system. This was seen to distort priorities and could result in poor alignment between actors. In Scotland, proposals to create a National Care Service (NCS) seek to address these issues by shifting accountability for social care to the Scottish government [20], although there are concerns about whether these proposals merely move the problem ‘upwards’ [21].

The economic context within which local systems are operating and seeking to implement more integrated approaches was seen as a particular challenge for realising the service transformation agenda. Again, these issues were reported to be most acute in Scotland and Italy, where prolonged periods of austerity following the global financial crisis of 2007/8 and a resultant focus on cost containment and efficiency savings was experienced as a constraint to what actors believed could be achieved locally. Similar experiences have been documented for other countries strongly affected by the financial crisis [6,22]. Importantly, the desire to control costs and make efficiency savings was seen as a key driver for pursuing the integration agenda in all three settings, including the Netherlands, where measures to control spending on health and social care have been subject to successive reform efforts [23].

Conversely, while the COVID-19 pandemic has placed major pressure on the health and social care systems in all countries, the pandemic was not considered as a catalyst for enduring novel ways of working. We found that the pandemic highlighted and exacerbated existing weaknesses within and across the health and social care systems in all three countries, confirming observations reported elsewhere [24]. Perhaps surprisingly, given that we sampled three countries with different approaches to driving integration, we did not identify particular integrated system governance *models* that showed themselves to be more (or less) resilient to recovering from the pandemic. Study participants highlighted several characteristics that have previously been documented as core to effective governance, such as the quality of local leadership, relationships with local government, openness and trust within and across collaborations [25,26,27,28].

Those implementing national policies reported being frustrated by the national government’s lack of insight into local realities, an observation also reported in other contexts [29]. In our study, this was linked to the lack of monitoring systems that adequately described the system as a whole. Indicators to measure the broader aims of integration were missing, and, as with funding, health, particularly acute care, data were seen to dominate in terms of both availability and use to measure performance. This was reported to contribute to further fragmentation and asymmetry between actors. Further, while acknowledging the need for performance monitoring, in all settings respondents wanted greater emphasis on the quality of care and patient experience.

Study participants suggested that for local integration efforts to be successful, there needed to be a consistent and long-term overall vision that is implemented at all levels of the system, from the centre to the periphery. We found that while Scotland has developed a long-term vision for integration as per its 2014 health and social care legislation, this was not seen to have translated into more effective collaboration locally. There is thus a need, at the national level, for greater integration across government departments to ensure consistency and credibility, and, ultimately, foster buy-in at local levels.

Our findings further point to the need for a tailored approach that creates a level playing field for those implementing integrated systems. Embedding integrated care is an incremental process and approaches need time to be tested and, if necessary, adapted. Time is also needed to foster meaningful and trusting relationships between partners and enable organisations to learn to function in new ways [4]. This was deemed especially important given that new ways of working were not always reported to result in immediately tangible benefits to participants. Evaluations of integrated care approaches in different settings have demonstrated that evidence of impact at population level is likely to be seen only after several years following implementation [30,31]. Supporting local innovation requires enduring commitment and long-lasting mandates to provide greater certainty over longer time-scales.

### Strengths and limitations

Governance is a multi-layered concept with different issues arising at the different tiers of the health (and social care) system [32]. Much of the previous work on integrated care has tended to focus on the meso-level, examining governance arrangements within organisations using different theoretical lenses, such as network governance [33], principal-agent theory [34], leadership theory [35], and/or conceptions of power [32]. In this study we were interested in how decision-making processes involves different levels in the different countries, and how this shaped integration and the interactions between levels. We used the TAPIC framework as a starting point to guide our analytical enquiry and ensure we cover important attributes of governance. TAPIC has been used elsewhere to describe and diagnose national-level policy challenges [8,36,37], with Exworthy et al. specifically applying the framework to assess the governance of integrated health and social care in England since 2010 [18]. However, while we found TAPIC helpful to conceptualise our approach, in particular in structuring the topic guides for interview, we found its operationalisation as an analytical lens for interview data challenging as identified themes intersected with multiple elements of TAPIC. Some of the identified themes mapped clearly onto TAPIC, such as accountability and capacity, but others did not, and we therefore decided to depart from the approach in the analysis of the interview data.

Further, undertaking data collection during the first and second waves of the COVID-19 pandemic posed considerable challenges to recruitment and the timing of interviews, as we did not want to add to the demands of study participants, all of whom were involved in their countries’ responses to the pandemic. This affected our ability to speak to experts contemporaneously; however, given COVID waves occurred at different times in different places, interviews are likely to have been undertaken at relatively similar phases of the pandemic.

Our study focused on the commonalities across studies sites and we prioritised breadth within each country over depth within individual study sites. This meant that we relied on a relatively small number of interviews in each study site and that it was only possible to touch on the complexities that exist within countries and study sites. To fully capture the governance complexities within study sites, and their interrelationships with the national level, would have required speaking with a larger number of people involved in the design, delivery and implementation of integrated approaches, along with the collection of additional data such as observations of meetings and the analysis of internal documents. This was however beyond the scope of this study.

### Implications for policy and practice

Our findings suggest several ways national governments can support local integration efforts better. Core will be the formulation of a unified, consistent, long-term national vision of health and social care integration across all tiers of the system. This needs to be combined with the flexibility and autonomy for local areas to be able to customise and adapt national policies to the local context [38].

Successful implementation requires stable local leadership to foster relationships and trust across the system. To support new ways of working may require additional resources, although, the economic context is likely to remain a challenge in any system. A continued focus on cost control and efficiency savings is likely to fundamentally constrain what can be achieved locally and to undermine the desired long-term transformation towards integrated delivery service [39].

Systematic monitoring needs to carefully balance the nature of ‘intelligence’ required to support and advance system transformation. Finally, the continued imbalance between health and social care, with the acute health care sector holding most of the power and financial resources, may mean that integration efforts continue to only occur ‘at the edges’ rather than leading to whole system change.

## Additional File

The additional file for this article can be found as follows:

10.5334/ijic.7610.s1 Appendix. Table A.1–Table A.2.
