# Supplementary material for: Governing Integrated Health and Social Care: An Analysis of Experiences in Three European Countries
Source: Int J Integr Care. 2024 Feb 7;24(1):9. doi: 10.5334/ijic.7610 (PMC10854466; doi:10.5334/ijic.7610)
Supplement: Appendix. — Table A.1–Table A.2. [file ijic-24-1-7610-s1.pdf]

(7) Appendix

Table A.1 Principles of health and social care systems in Italy, the Netherlands and Scotland

Country	Overall governance of health and social care	Provision and financing of health care and social care
Italy	Responsibility for **health care** governance is shared between the national government and the 20 regional governments. National government provides the legislative framework for health care; it sets the basic principles and objectives within which the National Health Service (SSN) operates, and monitoring the SSN. In collaboration with the regions, defines the national benefits package (*Livelli Essenziali di Assistenza,* LEA).<br><br>The national and regional levels of governance of the health care system are strategically aligned in Health Pacts, co-produced by national government and the regions every 3 years.<br><br>The regions are responsible for financing, organising and delivering health care, through local health authorities (ASLs). Regions are accountable to the electorate, and the national government for complying with the LEA standards. Where standards are not met or where regions, hospitals and ASLs financially underperform, they are sanctioned and can be subject to national government recovery plans.<br><br>ASLs are directly accountable to their regions.<br><br>**Social care** and social welfare services are overseen by municipalities (local authorities). Members of the municipality are elected.	**Health care** is provided through the National Health Service (SSN), with office-based GPs and paediatricians acting as gatekeepers to specialist care. The SSN is funded through national and regional taxation. Resource allocation to the regions is negotiated annually based on a capitation formula.<br><br>The SSN is regionally based. Within regions, health care delivery rests with geographically defined, local health authorities (ASL), which organise primary, secondary and tertiary health care by contracting with public and private hospitals and overseeing office-based GPs. The number of ASLs has declined, from 146 in 2010 to 99 in 2021, reflecting a broader trend towards centralisation of authorities at the regional level.<br><br>**Social care**: Municipalities have responsibility for supporting non-self-sufficient individuals on a means-tested basis, covering home help (housework and personal care) and nursing homes.<br><br>Health services that are delivered in nursing homes and in the community setting more broadly, such as home nursing services, home hospitalisation and programmed home care assistance by GPs, are funded by the SSN and provided by ASLs. In some Italian regions, municipalities have also delegated part of their social care duties to ASLs.
Netherlands	The health care system is based on regulated competition. **Health care** system governance is shared between the national government and the corporatist (self-governing) health sector.<br><br>The role of national government is largely restricted to overseeing and defining the rules for the health care system, including quality, accessibility, and affordability of health care. The national government defines the essential package of care that all health insurers must provide.<br><br>The health insurance market is overseen by the Dutch Health Care Authority (Nza), is responsible for compliance of insurers with the Health Insurance Act (Zvw) and sets payment rates.<br><br>Health insurers are regulated as commercial enterprises.<br><br>Governance of **social care** and preventive care that falls under the Long-term care Act (Wlz), are decentralised to local government (municipalities).	**Health care** financing is through mandatory health insurance, funded through income-related direct contributions, premiums, deductibles and government contributions for children, and complementary voluntary health insurance.<br><br>Insurers purchase and contract health services specified in the essential package of care. Health services are generally delivered by private providers, with office-based GPs acting as gatekeepers to hospital care. Financing of hospital services is based on activity using the Dutch version of diagnosis-related groups ('diagnosis treatment combinations', DBC).<br><br>**Social care**: Institutional care is typically provided by non-profit organisations while formal care at home is provided predominantly by private not-for-profit organisations. Financing of long-term care is through statutory social insurance; other social services, which do not come under health care, are financed through municipal budgets or general taxation. Municipalities are free to organise the care they provide.
Scotland	Responsibility for **health** and health services in Scotland rests with the Scottish Cabinet Secretary	**Health services** are delivered through the National Health Service (NHS), with office-based GPs acting

	for Health and Wellbeing. **Social care** is the responsibility of Local Authorities.<br><br>The 2014 Public Bodies (Joint Working) (Scotland) Act mandated NHS Boards and local authorities to integrate adult health and social care through the formation of Integration Authorities. From 2016, 31 Integration Authorities have had responsibility for commissioning integrated health and social care services from NHS Boards and Local Authorities.sss<br><br>The 14 NHS Boards are accountable to the national government. While local authorities are accountable to their electorate.<br><br>Legislation and planning are in place to introduce a National Care Service, making planning and delivery of social care accountable to central government.	as gatekeepers to specialist care. Health services provided through the NHS are financed primarily through general taxation. Health funding is allocated to the 14 NHS Boards who have a statutory responsibility for health planning and service delivery.<br><br>Local authorities provide or purchase almost all social care services in Scotland, with funding from general and local taxation (incl. transfers from NHS Boards), service user charges and other sources.<br><br>Integration Authorities hold joint or pooled budgets agreed by the NHS Health Board and Local Authority.

Table A 2 Key characteristics of the ten study sites in Italy, the Netherlands and Scotland

	Italy			The Netherlands				Scotland		
	**Azienda Zero (AZ), Veneto**	**Chronic care (Presa in carico del paziente cronico, PiC), Lombardy**	**Houses of health (Casa della salute, CdS), Emilia-Romagna**	**Buurtzorg**	**Rotterdam Stroke Service (RSS)**	**Sustainable coalitions**	**ZIO**	**Dumfries & Galloway, IJB**	**East Ayrshire, IJB**	**Highland, Integration Partnership**
**Origins**	Established in 2015 as part of regional care reform restructuring the Veneto health and social care system to centralise key functions previously undertaken by local health authorities.	Originated from a 2011 pilot in five local health authorities (ASLs) of bundled payments for integrated care for chronic conditions<br><br>Established in 2015 as part of wider regional reforms, which centralised health and social care oversight at regional level and introduced planning and commissioning agencies (ATS) responsible for planning and purchasing health and care services.	First proposed as 'medical homes' by national government (2007) to strengthen primary care and translated by Emilia-Romagna into regional plans, building on earlier (from 2004) efforts to establish primary care units.<br><br>Established in 2010 as part of wider regional reforms reorganising health and social care	Established as not-for-profit social enterprise in 2006 with one nursing team in the city of Almelo.	Established in 1997 as an informal arrangement between 1 hospital and a rehabilitation centre.	Launched in 2019 by the health insurer CZ in collaboration with large health care organisations, to support innovative approaches to service delivery, including integrated care.	Evolved from a diabetes disease management programme in 1996 into a care group in 2007 and ZIO primary care organisation in 2008.	Established in 2015 as a partnership between NHS Dumfries & Galloway and Dumfries & Galloway Council.	Established in 2015 as a partnership between NHS Ayrshire & Arran and East Ayrshire Council	Established in 2012 as partnership between NHS Highland and Highland Council.<br><br>First formal integrated care partnership to be established in Scotland.
**Scale/Population served**	Regional<br><br>Veneto population: ~4.9 million (2020)	Regional<br><br>Lombardy population: ~10 million (2020)<br><br>End 2019: 11% of 3 million people invited had joined the scheme; 8.5% (~260,000 people) had a care plan (PAI) drawn up.	Sub-regional<br><br>Emilia Romagna population: ~4.4 million (2020)<br><br>2018: 49% of the population covered by a CdS.<br><br>2020: 124 CdS established	National<br><br>2021: 950 teams ~ 14,700 nurses, serving a population of 86,400.<br><br>Nurses work in teams of 12 to provide care to 50-60 patients.	Regional<br><br>17 organisations across acute, primary and home care, arranged into 7 sub-chains connected to each participating hospital.<br><br>2021: ~4,000 patients treated.	National<br><br>CZ covers 21% of the Dutch population (~10.3 million in 2019).<br><br>2022: contracts with 4 hospitals, 2 mental health care organisations and 1 pharmacy chain.	Regional<br><br>Serves a population of ~170,000.<br><br>All GPs in region are affiliated with ZIO ≈ 84 GPs working in 54 practices.	Regional<br><br>Serves a population of ~150,000.	Regional<br><br>Serves a population of ~120,000.	Regional<br><br>Serves a population of ~320,000.
**Financing**	2019: AZ was allocated 6.7% of the region's health budget (≈€600 million)	ATS hold operating budget.<br><br>Payment to managing bodies is through the ATS using fixed-fee based on number of patients enrolled, who have signed a care pact and who have a care plan (PAI).<br><br>From 2019, incentive payments introduced for GPs to join scheme.	Department of primary care in each local health authority (ASL) holds operating budget.	Insurer pays health provider (Buurtzorg) for contracted services.	Insurer pays individual organisations within RSS for contracted services.<br><br>Piloting value-based health care across 4 organisations within RSS, with a single payment from insurer based on outcomes.	Insurer pays health provider for contracted services.	Insurer pays ZIO for contracted services.<br><br>Piloting pooled budget between health insurer and social care (municipalities) in four deprived neighbourhoods in Maastricht.	Pooled budget.<br><br>NHS Board and local authority provide funding and resources to the IJB. Chief Finance Officer develops funding requirements based on the Strategic Plan.		Pooled budget.<br><br>NHS Highland and Highland Council (local authority) agree to pay the other for the delegated services, determined by the delegating partner following negotiations.
**Leadership**	Led by Director General, supported by Committee which comprises the managing directors from each local health authorities (ASL, n=9) and the two hospital trusts in Veneto region.	ATSs are responsible for directing the implementation of PiC.<br><br>ATS are headed by a general manager appointed at the regional level.	Board comprising representatives from health care, municipal social care services and members of the public.<br><br>Organisational manager responsible for coordinating the CdS.	Led by Director, overseen by Supervisory board and Council of members.<br><br>Nursing teams are self-governing, with no team leaders or line managers.	General Board, made up of representatives from each participating organisation, ultimate decision-making body.<br><br>Executive Board responsible for day-to-day management. Comprises 7 members	'Top team' brings together CZ staff and provider organisations.<br><br>CZ led by Board of Directors, responsible for policy, strategy and results of CZ.	Executive team of Medical Director and Director of Operations, overseen by an independent supervisory board.<br><br>Supervisory board responsible for business operations and	IJB membership divided into non-voting and voting members. Voting members include non-executive member of the NHS Board and elected members from local authority and serve for 3 years. Non-voting including clinical, voluntary sector service users and carer representatives.<br><br>IJB appoint a chair and vice-chair from voting members: one from each party on a rotational		Highland Partnership Joint Monitoring Committee (HPJM) has<br><br>oversight of all delegated functions and the functions managed in conjunction with delegated functions.

	Italy			The Netherlands				Scotland		
	Azienda Zero (AZ), Veneto	Chronic care (Presa in carico del paziente cronico, PiC), Lombardy	Houses of health (Casa della salute, CdS), Emilia-Romagna	Buurtzorg	Rotterdam Stroke Service (RSS)	Sustainable coalitions	ZIO	Dumfries & Galloway, IJB	East Ayrshire, IJB	Highland, Integration Partnership
			Board and organisational manager prepare operational plans, support District Director, and promote participation of voluntary sector and public.		from participating organisations. No set length of service, many on boards since RSS established.<br>Managing Director supports the Executive Board.<br>Local chain coordinators support the sub-chains.	Management team supports Board of Directors. Supervisory board and Council of Members oversees activities of Board of Directors.<br>Each provider organisation governed by its own leadership team.	organisation performance. Comprises three members that serve four-year terms.	basis. Hold post for 2 years.<br>Chief Officer appointed to act as a single senior point of overall strategic and operational advice to the IJB & Chief Finance Officer.		Committee comprises elected members nominated by local authority and by NHS Board, plus officers and staff of either organisation, third sector, carer/service user representatives, among others.<br>Jointly chaired by Chair of NHS Highland Board and the Chair of Highland Council's Health, Social Care and<br>Wellbeing Committee.
**Accountability**	AZ is accountable to regional government, and its functions are specified by regional legislation.<br>As a public body all decisions must be made publicly available; published on website.<br>Public and private secondary care providers accountable to AZ.	ATS are accountable to the regional government.<br>Managing bodies are accountable to ATS for providing services as set out in care plans (PAI), and to patients.<br>Implementation of PAI is attached to financial performance.	Department of primary care is accountable to ASL. Responsible for managing the interface with other departments at district level and liaises with all stakeholder involved in CdS.<br>Municipalities accountable to local electorate.	Buurtzorg accountable to health insurers for the delivery of services in accordance with contract.	Health care providers accountable to health insurer in accordance with contract.<br>Participants accountable to General Board to deliver care in line with the chain protocols.<br>Agreements between participating organisations along the care chain, including tasks, responsibilities, criteria for patient transfers and information sharing set out in chain protocols.	Sustainable coalitions between CZ and provider are formalised in legally binding contracts.<br>CZ accountable to national government.<br>Health providers accountable to CZ in accordance with contract.	ZIO is ultimately accountable for the delivery and performance of the care group.<br>Care providers are jointly accountable with ZIO to insurer for delivering care in accordance with care protocols.	IJB has operational oversight of the NHS Board and local authority.<br>NHS board and local authority are accountable to IJB for the delivery of services in accordance with the Strategic Plan.<br>Chief Officer and Chief Finance Officer accountable to the Chief Executive of both parties.<br>Health and Care Governance Group are chaired by Chief Officer, responsible for oversight of health and care governance.<br>NHS Board accountable to Scottish minsters and Scottish Parliament<br>Local authority accountable to the electorate.		Lead agency has operational responsibility for delivery of delegated services, accountable to HPJM.<br>NHS Highland accountable to Scottish minsters and Scottish Parliament<br>Highland Council (local authority) accountable to electorate.
**Monitoring & reporting Evaluation**	AZ legally required to establish an independent evaluation body to monitor its performances.<br>From 2020 required to publish a yearly report.	Performance of managing bodies and providers monitored by ATS on four domains; reaching a threshold number of enrolled patients, case-mix, delivery of care plans (PAI) and outcomes.	Monitoring reports published on the regional government's website.<br>Report on organisational aspects; number of CdS, composition of Board, range of activities carried out, finances.	Nursing teams have to meet operational targets incl. productivity targets, patient satisfaction and number of clients.<br>Performance data published on internal IT system (BuurtzorgWeb), not made public.	General Board meets annually to review progress and agree targets.<br>Annual quality standards, published in annual report online.<br>Three core set of indicators: (1) national benchmark for hospitals (DICA/DASA); (2) RSS indicators for follow-up institutions; (3) RSS indicator set for stroke aftercare.<br>Audits of all participating organisations every 2	Sustainable coalitions are evaluated and monitored against a self-developed approach (i.e., Impact Method) focusing on short, medium, and long-term outcomes, their drivers, and their reach.<br>Findings have not been made public.	Supervisory board holds an annual performance review with executive team to review collaboration and accounts, approve budgets and activity plans.<br>Quality-indicators for each care programme developed by multidisciplinary working groups based on national quality indicators.<br>Committed to continuous monitoring and evaluation, part of which provides mirror	Meeting minutes, audits, papers, and financial accounts published on website.<br>Annual performance report, documenting progress on nine national health and social care wellbeing outcomes (East Ayrshire additionally reports on national outcomes for children and justice (3 indicators in each) published on website.<br>Independently contracted services audited by the Care Inspectorate.<br>Annual audits of IJB & Highland Council published by Audit Scotland.		

	Italy			The Netherlands				Scotland		
	**Azienda Zero (AZ), Veneto**	**Chronic care (Presa in carico del paziente cronico, PiC), Lombardy**	**Houses of health (Casa della salute, CdS), Emilia-Romagna**	**Buurtzorg**	**Rotterdam Stroke Service (RSS)**	**Sustainable coalitions**	**ZIO**	**Dumfries & Galloway, IJB**	**East Ayrshire, IJB**	**Highland, Integration Partnership**
					years by the RSS.		information to care group partners.			

Note: Authors' compilation based on country reports (available upon request).
